# Homeostatic T Cell Proliferation after Islet Transplantation

**DOI:** 10.1155/2013/217934

**Published:** 2013-07-22

**Authors:** Paolo Monti, Lorenzo Piemonti

**Affiliations:** San Raffaele Diabetes Research Institute, San Raffaele Scientific Institute, Via Olgettina 60, 20132 Milan, Italy

## Abstract

Pancreatic islet transplantation in patients with type 1 diabetes mellitus is performed under immunosuppression to avoid alloreactive T cell responses and to control the reactivation of autoreactive memory T cells. However, lymphopenia associated with immunosuppression and T cell depletion can induce a paradoxical expansion of lymphocyte subsets under the influence of homeostatic proliferation. Homeostatic T cell proliferation is mainly driven by the IL-7/IL-7 receptor axis, a molecular pathway which is not affected by standard immune-suppressive drugs and, consequently, represents a novel potential target for immuno-modulatory strategies. In this review, we will discuss how homeostatic T cell proliferation can support autoimmunity recurrence after islet transplantation and how it can be targeted by new therapeutic approaches.

## 1. Introduction

Type 1 diabetes mellitus is a chronic autoimmune disease resulting from the selective destruction of insulin producing beta cells in the islet of Langerhans. Islet transplantation is clinically indicated to replace the insulin producing beta cell mass in patients with type 1 diabetes mellitus and therefore represents a potential cure for the disease [1, 2]. Islet transplantation in patients with type 1 diabetes represents a complex immunological challenge. In fact, donor islets express allogeneic histocompatibility antigens, and therefore recipients are treated with immunosuppression to avoid graft rejection. In addition, donor islets express beta cell antigens which are targeted by T cells and B cells during the autoimmune process. Glutamic acid decarboxylase 65 (GAD65), insulinoma-associated protein 2 (IA2), and (pro)insulin appear to be highly antigenic in humans both for T cells [3] and B cells [4] during the natural history of type 1 diabetes. Beta cell replacement into a recipient with preexisting T cell and B cell responses to autoantigens represents a rechallenge of the recipient immune system and can result in autoimmunity recurrence postislet transplantation [5, 6]. Unlike allogeneic T cell responses, autoimmunity recurrence is difficult to control with standard immunosuppression and therefore poses an additional set of therapeutic obstacles for a successful long-term function of islet allografts. How memory autoreactive T cells expand under immunosuppression after islet transplantation is largely unknown. Memory autoreactive T cells can be more resistant both to inhibition by immunosuppressive compounds and to regulation by regulatory T cells. In recent years, a growing body of evidence suggests that lymphopenia associated with immunosuppression and immunodepleting agents can play an important role in expanding memory autoreactive T cells. The immune system can sense the lymphocyte loss and respond with a vigorous cytokine-mediated expansion of remaining lymphocytes, a process known as homeostatic proliferation [7, 8]. Homeostatic proliferation can involve effector T cells but also regulatory T cells and B cells. Homeostatic proliferation of effector T cells, including autoreactive T cell clones, has been shown to exist in patients undergoing islet transplantation. In this review, we will discuss the role and mechanisms of homeostatic lymphocyte expansion in islet transplantation. In addition, we will discuss whether standard immunosuppressive drugs can keep homeostatic proliferation under control or whether we would need to develop alternative strategies to specifically target homeostatic proliferation.

## 2. Autoimmune Memory in Type 1 Diabetes

Activation of autoreactive B cells and T cells specific for islet beta cells precedes clinical onset of diabetes. To date, immune markers for type 1 diabetes have been primarily centered on the presence of autoantibodies to beta cell antigens, and measurement of these autoantibodies has been shown to be useful for the prediction of type 1 diabetes. In contrast, the detection of circulating T cells specific to islet autoantigens has been inconsistent in its diabetes specificity mainly because islet specific T cells are commonly found in all individuals, including those without any sign of beta cell specific autoimmunity. The crucial difference between healthy and type 1 diabetic subjects is that autoreactive T cells in patients with type 1 diabetes display an antigen experienced phenotype whereas in healthy individuals they display a naïve phenotype. Three observations were key to support this conclusion. First, autoreactive T cells in patients with type 1 diabetes proliferate in response to beta cell antigens in the absence of costimulatory signals required to trigger proliferation of autoreactive T cells from healthy controls [9]. Second, a significant proportion of autoreactive T cells in patients with type 1 diabetes express the effector and memory marker CD45RO, whereas almost all autoreactive T cells from healthy individuals are CD45RO negative [10, 11]. Third, autoreactive T cells from patients with type 1 diabetes have shorter telomere length than autoreactive T cells from healthy controls. In T cells, telomeres undergo 50–100 base pair shortening in each cell division. Similar to memory T cells specific for tetanus toxoid, T cells specific for the beta cell associated antigens GAD65 and insulin have shorter telomeres, both in patients at disease onset and in prediabetic autoantibody positive patients suggesting an in vivo history of antigen stimulation and cell divisions [11]. The development and the persistence of a pool of autoreactive T cells with a memory phenotype represent the pathological base for autoimmunity recurrence after islet transplantation.

## 3. Recurrence of Autoimmunity after Islet Transplantation

Patients with long-term type 1 diabetes who received beta cell transplants still have autoimmune memory with the capacity to destroy islets. Seminal is the evidence that identical twin transplants performed by David Sutherland in which pancreas a segment from an unaffected twin was transplanted in the twin with long-term type 1 diabetes in the absence of immunosuppression, and it resulted in the loss function of grafted beta cells and in the development of insulitis, reminiscent of what is seen at diabetes onset [12, 13]. In addition to this, there is a good evidence to indicate that transplantation of isolated allogeneic islets can cause relapse of autoimmunity in a small but significant proportion of patients. Occasionally transplanted patients had dramatic rises in islet autoantibodies within few weeks after transplant. This rise usually occurred without any sign of alloimmunity [14]. Weaker immune suppression regimens such as MMF plus 1,25 dihydroxy vitamin D3 were more frequently associated with autoantibody rises with or without evidence of an alloreaction [15]. Although recipients who had a posttransplant antibody increase showed similar initial performance of islet grafts, a significantly lower long-term graft survival was observed in those patients as compared to patients without an increase in islet autoantibodies [5]. T cell responses to islet autoantigens are often increased after islet transplants [16]. These conclusion were drawn either by analyzing T cell proliferation in response to stimulation with islet specific proteins in vitro or by staining peripheral blood mononuclear cells with islet specific fluorescent HLA multimers. Observations of autoimmunity recurrence as assessed by autoantibodies and occasionally T cells have also been reported following allogeneic pancreas transplants under immune suppression. In the clinical setting of simultaneous pancreas-kidney transplantation, Vendrame and colleagues clearly showed the presence of pretransplant autoreactive CD4+ T cell clones and reappearance of the same clones after depletion with thymoglobulin plus daclizumab [17]. These cases appear to be less frequent [18] and of less proven clinical relevance [19]. The infrequency of these cases suggests that immune suppression in the context of whole pancreas transplant was usually sufficient to keep autoimmune memory under control. It is unclear whether this is due to the immunosuppressive regimen (currently conditioning through depletion and maintenance with FK506, MMF, and often steroids) or the nature of whole organ transplant.

Although associations with reduced graft function have been reported, it is not proven that autoimmunity recurrence equals to autoimmune mediated destruction of grafted beta cells. One important issue is the donor/recipient HLA mismatch. TCR specificity of preexisting autoreactive T cells is for a combination of specific antigens in a recipient MHC context. Studies performed with fluorescent HLA multimers (HLA of the recipients) clearly showed that the autoimmune disease recurrence is directed to the islet antigens from the graft presented to T cells in the context of recipient antigen presenting cells, somewhat analogous to allogeneic antigen presentation through indirect allorecognition pathways [16]. In the setting of whole pancreas transplant, TCR analysis and identification of Vbeta sequences in tetramer binding T cells clearly suggest a persistent memory response expanded by preexisting progenitors [20]. Although these data strongly suggest that autoimmunity recurrence is caused by pretransplant memory clones, how recipient CD8+ cytotoxic clones can recognize and destroy donor HLA mismatched beta cells remains to be elucidated.

## 4. Homeostatic T Cell Proliferation in the Generation and Maintenance of Memory Autoimmunity

**Figure 1:**
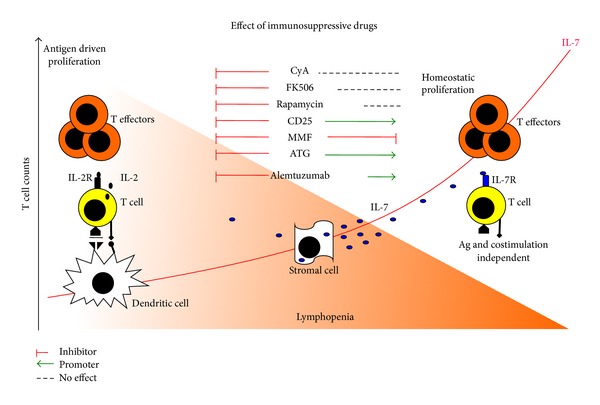
A model comparison of antigen driven versus homeostatic T cell proliferation. In conditions of immune competence and steady-state circulating lymphocyte counts, T cell proliferation can occur in an antigen-specific manner. MHC-peptide antigen complex is recognized by a specific T cell receptor on the T cell surface. Antigen-activated T cells express CD25 and produce IL-2 in an autocrine manner. Low levels of IL-7 are necessary for T cell survival. Reduction of T cell number is accompanied by increased IL-7. Proliferation of T cells relies on homeostatic mechanisms. T cells express the IL-7 receptor stimulated by supraphysiological levels of IL-7. Proliferation is antigen and co-stimulation independent and generated effector cells with characteristics that are remarkably similar to those of antigen expanded T cells. All immunosuppressive drugs inhibit antigen-specific T cell proliferation. In contrast, during homeostatic proliferation only MMF can inhibit T cell expansion, whereas cyclosporine A, FK506, and rapamycin have no effect and anti-CD25 monoclonal promotes IL-7 mediated proliferation.

A background of autoreactive T cells with an antigen experienced memory phenotype remains in the immune system of patients with type 1 diabetes and can be reactivated upon antigen rechallenge through islet transplantation. Long-term persistence of memory T cells in humans is achieved by a combination of two processes: first, the long-term survival of individual cells, and second, periodic cell division to balance attrition through cell death. Studies performed by human T cell radio labeling (^2^H_2_O, ^2^H glucose) and mathematical modeling estimated a median turnover rates of naïve CD4+ and CD8+ T cells to be approximately 4 and 6 years, respectively, and of 6 and 8 months for CD4+ and CD8+ memory T cells [21]. Among CD4+ memory T cell subpopulation, the turnover rate appeared to be faster in CCR7− effector memory T cells as compared to CD45RO+CCR7+ central memory T cells [22]. The concept that immunological memory is maintained by proliferating rather than quiescent long lived memory T cells raises the question of how memory T cells survive and proliferate to a sufficient rate for years with low or absent antigenic stimulation. Naïve T cells rely on survival signals through contact with self-peptide-loaded MHC molecules plus stimulation with homeostatic cytokines IL-7 and IL-15 [23, 24]. Conversely, antigen-experienced T cell populations are typically MHC independent, and they survive and undergo periodic homeostatic proliferation through IL-7 and IL-15, whose receptors are highly expressed on memory T cells [25, 26] (Figure 1).

Steady-state homeostatic T cell proliferation can be dramatically increased in conditions of lymphopenia. The immune system can sense T cell loss and respond with a vigorous cytokine mediated expansion of remaining T cells driven by supraphysiological concentrations of IL-7 [27]. Unlike steady-state homeostatic proliferation, T cells undergoing “acute” homeostatic proliferation develop properties that are remarkably similar to those of antigen expanded T cells, including effector/memory phenotype and effector function [28]. As a consequence, homeostatic T cell proliferation is suggested to promote T cell-mediated autoimmunity. In fact, autoreactive T cells that escape negative selection in the thymus have typically a low affinity for the cognate self-antigens. Due to insufficient TCR signals, autoreactive T cells do not reach the threshold for upregulation of the IL-2 receptor alpha and autocrine production of IL-2, and this represents a barrier to their activation and expansion in most individuals. During homeostatic proliferation, however, common gamma chain signals for T cell proliferation are provided by IL-7 and IL-15, and quiescent autoreactive T cell clones can undergo expansion and activation despite low TCR signals [29].

Homeostatic T cell proliferation has been implicated in the expansion of autoreactive T cells that causes islet autoimmunity. In the nonobese diabetic (NOD) mouse model, a chronic condition of T cell lymphopenia has been associated with increased homeostatic proliferation and beta cell autoimmunity [30]. Moreover, IL-7 accelerates diabetes in NOD mice, while blockade of the IL-7R can reverse diabetes in the same model [31, 32]. An increased sensitivity of splenic T cells to IL-7 was observed in two models of allogenic islet transplantation in NOD recipients treated with anti-CD45RB and CD154 antibodies [33] as well as under an “Edmonton like” protocol (tacrolimus, rapamycin, and anti-CD25 antibody) [34]. 

Reduced circulating T cell counts have been recently reported also in patients with type 1 diabetes [35]; however, no signs of increased circulating IL-7 or increased homeostatic T cell proliferation have been reported in those patients. In humans, polymorphisms of the IL-7R alpha were associated with an increased risk of developing the disease [36], and nonenzymatic glycation of the IL-7R alpha in patients with established diabetes and hyperglycemia appear to affect IL-7 signaling [37].

## 5. Homeostatic Proliferation after Islet Transplantation

Islet transplantation is performed under immuno-suppression in which lymphopenia is a common side effect. Induction with depleting agents such as ATG and alemtuzumab (anti-CD52) can substantially influence the severity of lymphocyte loss and possibly the rate of cell cycling during reconstitution. Different immune-suppressive compounds, the rate of cell cycling, and different needs for homeostatic proliferation can influence the relative composition of the lymphocyte compartment after reconstitution. 

### 5.1. Autoreactive T Cells

Autoreactive T cells can homeostatically expand after islet transplantation [38]. Increased concentrations of IL-7 and IL-15 have also been found in patients with type 1 diabetes after islet transplantation [38]. This may reflect the homeostatic T cell response to the relative lymphodepletion associated with the transplant immuno-suppressive therapy. In fact, even with a relatively modest reduction in circulating lymphocyte numbers (as using an induction regimen with the nondepleting anti-CD25 monoclonal antibody daclizumab and maintenance therapy with rapamycin and FK506) increased lymphocyte turnover (Ki-67+ cycling T cells) is seen early after transplantation. Homeostatic T cell proliferation occurs in both CD4+ and CD8+ T cells, and proliferating cells display CD45RO+ memory phenotype and IFN-gamma production. Antigen specificity of cycling T cells is broad and includes both memory T cells specific for viral antigens (Influenza matrix protein) and the beta cell autoantigen GAD65. This is an example of how an immunosuppressive treatment designed to control allo- and autoimmunity can activate compensatory mechanisms which favors the emergence of memory T cell responses and paradoxically promotes autoimmunity recurrence. 

### 5.2. Alloreactive T Cells

Alloreactive T cells are expanded after lymphocyte depletion therapies with alemtuzumab or ATG, in some cases far exceeding pretransplant levels [39, 40]. Alemtuzumab treatment has been shown to preferentially expand effector-memory T cells in renal transplant recipients whereas induction with ATG expands both effector-memory and central-memory T cells subsets. Of note, episodes of rejection were described to be preceded by a significant increase in effector-memory T cells [41]. 

### 5.3. CD4+CD25+FOXP3+ Regulatory T Cells

CD4+CD25+FOXP3+ regulatory T cells (Treg cells) are distinguished by low expression of the IL-7R alpha as compared to conventional T cell subsets [42, 43]. In vitro studies of IL-7 signaling showed that Treg cells can sense IL-7 at a concentration 100–1000 fold higher than conventional naïve and memory T cell subsets [44]. In steady state indeed, Treg cells constitutively express the IL-2 receptor alpha chain (CD25), and their survival is largely IL-2 dependent. Moreover, another hallmark of Treg cells is their anergy. Overcoming anergy in Treg cells is achieved via a combination of strong TCR and IL-2 signaling [45]. IL-7 plus strong TCR signaling can induce proliferation of Treg cells with a naïve phenotype whereas memory Treg cells remain anergic [44]. Taken together, these pieces evidence suggest that Treg cells are less susceptible to homeostatic proliferation than conventional T cells. This may in part explain why there is an impairment of the Treg compartment after recovery from lymphopenia that has been shown both in animal models and in humans. For example, in NOD mice sublethal irradiation delayed the onset of hyperglycemia, but reconstitution by adoptive transfer of splenocytes precipitated disease onset despite the increased content of Treg in the pancreas and draining lymphoid tissue. Similar results were also observed when purified CD25+ cells where infused after radiation [46]. In humans, patients receiving allogeneic hematopoietic stem cell transplantation show a high rate of Treg proliferation; however, the majority of these cells die by Fas-mediated apoptosis. Apparently, Treg cells expand faster than conventional T cells, leading to their initial accumulation both in the periphery and in the bone marrow [47]. Prospective monitoring of reconstitution however reveals that Treg cells decline after the expansion and the T cell compartment is characterized by a significant Treg deficiency after reconstitution [48]. Comparison of different induction therapies used in islet transplantation showed a different impact on the relative frequency of Treg cells after reconstitution. Treg cells remained stable after ATG treatment, whereas a sharp increase was observed starting from the first month after alemtuzumab induction and returned to baseline after 6 months. In contrast, the frequency of FOXP3+ T cells declines to almost undetectable level after induction with the nondepleting anti-CD25 antibody daclizumab. In terms of absolute counts, however, all induction strategies induced a sustained drop in the first month, and there was no recovery of FOXP3+ T cell counts for at least 12 months of observation [49].

### 5.4. B Cells

B cells do not express the IL-7 receptor alpha; however, they recover after depletion both by the novo lymphopoiesis (the recruitment of B cell precursors from the bone marrow), as well as by homeostatic proliferation in the periphery. For B cells, the cytokine BlyS (also known as B-cell activating factor) is the main homeostatic cytokine for both naïve and memory B cells, In addition, BlyS sustains survival of plasmablasts and plasma cells [50]. Importantly, BlyS functions as the main sensor of B cell lymphopenia, analogous to the role of IL-7 for naïve T cells [51]. Circulating BlyS levels are increased after B cell depletion and support survival of transitional B cells [52]. A growing body of evidence suggests that the therapeutic effect of B cell depletion derives from its long-term effect on B cell reconstitution in addition to more immediate deletion of pathogenic B cells. B cell depletion was associated with prolonged expansion of CD24^high^CD38^high^ transitional B cells and delayed reemergence of memory B cells [53]. In some cases, memory B cells did not appear for several years [54]. In a nonhuman primate model of islet transplantation using induction with rituximab and ATG followed by maintenance with sirolimus monotherapy, a high ratio of transitional B cells to memory B cells was associated with allograft acceptance in the absence of immunosuppression [55]. At present, it is unclear whether the islet outcome improvement is mediated by the relative absence of memory B cells or by the presence of transitional B cells.

## 6. Effect of Standard Immunosuppression on Homeostatically Expanding T Cells

The majority of immunosuppressive drugs used in islet transplantation protocols are designed to inhibit specific pathways of antigen specific T cell activation (Figure 1). FK506 and cyclosporine A, when complexed to their respective immunophilins, form a ternary complex with calcineurin, a calmodulin (calcium modulating protein) dependent serine/threonine phosphatase, causing its inactivation. This results in the inhibition of the ability of calcineurin to dephosphorylate the cytoplasmic subunit of the nuclear factor of activated T cells (NFAT), thereby blocking its translocation to the nucleus, which is required for the transcriptional activation of cytokine genes, the most important of which is IL-2 [56]. Rapamycin is widely used in islet transplantation protocols. The rapamycin-FKBP complex binds directly to mTOR and blocks its function. By interfering with the function of mTOR, sirolimus inhibits the mTOR mediated signal transduction pathways resulting in the arrest of the cell cycle in the G1 phase in various cell types. This includes the signal transduction pathway of IL-2 [57]. Cyclosporine A, FK506, and rapamycin can efficiently block the IL-2 pathway. However, they were quite ineffective in controlling the IL-7 mediated homeostatic proliferation [38]. IL-7 is constitutively produced by stromal cells located in the bone marrow, and circulating IL-7 levels are not negatively affected by the administration of these drugs [58]. IL-7 signalling depends on the engagement of the IL-7R alpha and the common gamma chain on the T cell surface that activates STAT5 and AKT [59]. Although signalling of IL-2 and IL-7 appears to be similar, IL-7 has a potent antiapoptotic and trophic effect on T cells, suggesting that different pathways which do not involve the rapamycin sensitive mTOR complex are activated. 

The nondepleting anti-CD25 monoclonal antibody daclizumab was specifically designed to prevent the formation of the high affinity IL-2R complex and to block the IL-2 signalling [60]. Surprisingly, T cells treated with daclizumab become more sensitive to IL-7. The availability of the common gamma chain shared by IL-2 and IL-7 receptors represents a limiting factor for cytokine signalling. When the formation of the IL-2 receptor is inhibited by daclizumab, there is more common gamma chain available for complexing with the IL-7R alpha, resulting in an increased T cell sensitivity to IL-7 [61]. Different from the other compounds, mycophenolate mofetil (MMF) is a cytostatic drug that blocks DNA synthesis by inhibiting the enzyme inosine monophosphate dehydrogenase which is required for purine synthesis during cell division [62]. MMF acts directly on DNA synthesis downstream to all cytokine pathways that induces proliferation. Patients undergoing islet transplantation that switched from rapamycin to MMF for adverse side effects of rapamycin showed a decrease of cycling Ki-67+ T cells to the level that was compatible with the pretransplant levels [38]. Of the immunosuppressive compounds tested, MMF was the only effective inhibitor of IL-7 mediated T cell proliferation.

## 7. Targeting IL-7 Mediated Homeostatic T Cell Proliferation

The IL-7/IL-7R pathway is a potential therapeutic target to control autoimmune cell expansion and proliferation. Biological agents targeting specific cytokine pathways such as TNF alpha and IL-1 have had a considerable impact on the management of autoimmune diseases in more recent years. So far, no efforts have been made to target IL-7 in humans. Two recently published studies suggest that blocking the function of IL-7 could modulate T cell autoreactivity in the NOD mouse model [31, 32]. In these studies, administration of IL-7R blocking antibodies once or twice a week prevented the onset of the disease and normalized blood glucose levels in 50–85% of mice after diabetes onset. Whether this approach can be used to control autoimmunity recurrence in islet transplantation models has not been addressed yet. As with many cytokine receptors, a soluble form of the IL-7 receptor *α* chain (sCD127) has been identified [63]. It is derived both from alternative splicing and by release of membrane bound sCD127. The production of sCD127 is in part determined by regulation of transcription and mRNA processing, especially splicing, and this is affected by polymorphisms within the CD127 gene. Four common CD127 haplotypes have been described [64]. Haplotype 2 is identified by a T allele in exon 6 (rs6897932). This single nucleotide polymorphism (SNP) is associated with reduced exon splicing and lower production of mRNA encoding soluble IL-7Ra in healthy control individuals. In contrast, transcripts that skip exon 6 (“C” allele of rs 6897932) confer susceptibility to type 1 diabetes and encode a soluble form of CD127 (sCD127), potentially altering the production of sCD127 and IL-7R signaling in T cells [65]. sCD127 binds to and inhibits the biological activity of IL-7 and is therefore a potentially useful endogenous regulator of homeostatic T cell proliferation [37].

Another biological compound, the soluble HIV Tat protein was shown to downregulate IL-7R signalling in T cells [66]. Tat is a 15 kDa viral protein secreted by HIV infected cells which can be found in the supernatant of in vitro infection studies as well as in the serum of HIV infected individuals. Interestingly, purified Tat proteins downregulate the IL-7R on T cells from healthy donors. Soluble Tat proteins are taken up by CD8 T cells and enter the cytoplasm through a process that requires endosomal acidification. Once in the cytoplasm, Tat translocates to the inner leaflet of the cell membrane, where it interacts with the cytoplasmic tail of CD127, inducing receptor clustering and removal from the cell surface in a microtubule-dependent manner. Finally, Tat appears to direct CD127 to the proteasomes for degradation.

## 8. Concluding Remarks

Autoreactive T cell expansion in type 1 diabetes and after islet transplantation is traditionally considered a consequence of hyperstimulation of the immune system in an inflammatory milieu. In line with this concept, autoreactive T cell expansion has been therapeutically approached with standard immunosuppressive drugs. However, this approach did not provide the expected results. Evidence that lymphopenia driven homeostatic T proliferation can be the driving force of autoreactive T cell expansion in islet transplantation questions this concept. Homeostatic T cell proliferation is active in conditions of immuneinsufficiency and is largely ignored by standard immunosuppression. To achieve an efficient control of autoimmunity recurrence after islet transplantation, in addition to T cell depletion and inhibition of antigen specific T cell activation, specific targeting of homeostatic pathways could be beneficial to achieve the control of autoreactive T cell expansion and activation.
